# Trade-off analysis between g_m_/I_D_ and f_T_ of GNR-FETs with single-gate and double-gate device structure

**DOI:** 10.1038/s41598-024-59908-5

**Published:** 2024-05-03

**Authors:** Md Akram Ahmad, Pankaj Kumar, Bhubon Chandra Mech, Jitendra Kumar

**Affiliations:** 1 Department of Electronics Engineering, Indian Institutes of Technology, Dhanbad, 826004 India; 2 Department of Electronics and Communication Engineering, Graphic Era University, Dehradun, 248002 India; 3 Department of Electronics Engineering, Defence Institute of Advanced Technology, Pune, 411025 India

**Keywords:** Graphene nanoribbon (GNR), Analog/RF, Field-effect-transistor (FET), Trade-off analysis, Non-equilibrium Green’s function (NEGF), Materials science, Nanoscience and technology

## Abstract

This study examines the operational parameters of field-effect transistors (FETs) using single-gate (SG) and double-gate (DG) graphene nanoribbons (GNRs) within the analog/RF domain. A detailed exploration is conducted through an atomistic *p*_z_ orbital model, derived from the Hamiltonian of graphene nanoribbons, employing the nonequilibrium Green’s function formalism (NEGF) for analysis. The atomic characteristics of the GNRFETs channel are accurately described by utilizing a tight-binding Hamiltonian with an atomistic *p*_z_ orbital basis set. The primary focus of the analysis revolves around essential analog/RF parameters such as transconductance, transconductance generation factor (TGF), output resistance, early voltage, intrinsic gain, gate capacitance, cut-off frequency, and transit time. Furthermore, the study assesses the gain frequency product (GFP), transfer frequency product (TFP), and gain transfer frequency product (GTFP) to evaluate the balance between transistor efficiency, gain, and cut-off frequency. The research outcomes indicate that double-gate GNRFETs exhibit superior analog/RF performance in comparison to their single-gate counterparts. However, both types of devices demonstrate cut-off frequencies in the gigahertz range. The extensive data presented in this study provides valuable insights into the characteristics of SG and DG GNRFETs, particularly in terms of the figure-of-merit (FoM) for analog/RF performance, offering a comprehensive analysis of the trade-offs in analog applications. In addition, the analysis has been extended be performing a high-performance hybrid 6T static random-access memory (SRAM) to get the impact in their circuit level variation as well as improvement in their circuit performance.

## Introduction

In recent years, there has been a significant reduction in the size of transistors, transitioning from the micrometer to the nanometer scale in line with Moor’s Law^1,2^. Despite this progress, the increasing demand for advanced electronic devices has posed substantial challenges to the limitations of silicon-based transistors in terms of size. The primary challenges include short-channel effects (SCE), parameter fluctuations due to parasitic impacts, process variations, and dopant randomness. Researchers have actively engaged in exploring alternative materials to address these limitations. Graphene has emerged as a prominent candidate due to its widespread availability and cost-effectiveness^3^.

Graphene, a single-layered carbon atom structure, has garnered attention for its exceptional properties, making it a compelling material for the future generation’s semiconductor devices. These properties include remarkable thermal conductivity, high saturation velocity, flexibility, robust mechanical strength, and high carrier mobility^4–8^. Graphene’s outstanding mobility characteristics make it particularly suitable for applications in flexible and radio frequency (RF) devices^9,10^. Additionally, its ambipolar transport characteristics offer unique advantages for electronic device applications^11,12^. Nevertheless, graphene nanoribbons (GNRs) exhibit greater promise compared to graphene, given that the latter is a material with zero bandgap, making it unsuitable for switching applications. GNRs, which are elongated cut-out strips of graphene sharing electronic characteristics with semi-infinite graphene sheets, are generally presumed to possess finite nanometer dimensions. The bandgap of GNRs can be deliberately manipulated, presenting them as potential candidates for nano-electronic devices. Utilizing advanced patterning techniques like e-beam lithography, a slender strip of one-dimensional GNRs can be crafted from a 2D graphene monolayer^13–17^. GNRs come with two primary edge shapes—armchair GNRs (AGNRs) and zigzag GNRs (ZGNRs)—depending on their edge orientation. ZGNRs consistently exhibit metallic properties, while AGNRs can be categorized into three families: 3*p*, 3*p* + 1, and 3*p* + 2. The 3*p* and 3*p* + 1 families act as semiconductors, while the 3*p* + 2 family displays metallic characteristics. The bandgap significantly influences the performance metrics of devices, underscoring the crucial role of a band structure approach in the design and manufacturing of nanoelectronics^18^. Typically, the GNR-FET represents a standard field-effect transistor (FET) device in which a GNR channel is incorporated between the drain and the source. The channels within GNR-FETs exhibit remarkable sensitivity, making them applicable across a broad spectrum of practical uses. What sets GNR-FETs apart from conventional FET technologies is their distinctive switching capability between p and n channels^19^. It has been observed that the subtle unintentional doping in graphene samples, resulting from the deposition of the top-gate stack, counteracts the compact nature of the device^20^. Some researchers have determined the mobility of charge carriers, considering the minimal carrier concentration in a specific channel based on its length. Consequently, they strongly advocate for the GNR-FET as a promising alternative in the post-Silicon era of the semiconductor industry^21^. Following extensive research, top-gated GNR-FETs with a mobility range of 6000–7000 cm^2^ V^−1^ s^−1^ have been developed by optimizing the high-k dielectric material. Notably, the mobility of GNR-FETs surpasses that of traditional Si-based FET devices^22–24^. Additionally, reports indicate that the transconductance and transit frequency of GNR-FETs exhibit higher values compared to those of similarly sized CMOS structures^25^. Consequently, the GNR-FET holds tremendous potential in the field of microwave and radio frequency (RF) devices.

As electronic devices continue to shrink into the nanometer range, maintaining satisfactory analog/RF performance parameters while minimizing power consumption poses an escalating challenge^26^. Recent studies have delved into assessing the analog/RF performance of graphene nanoribbon field-effect transistors, exploring the impact of diverse factors on these parameters. These factors encompass distinct dielectrics^27^, gate length, gate oxide material, and gate oxide thickness^28^, along with considerations of the underlap effect^29^ and vacancy defects^30^. Another avenue to enhance analog/RF performance involves the adoption of triple-material gates^31^. Nevertheless, there remains considerable room for further research to optimize analog/RF parameters, and the capability to interchange and fine-tune these parameters is crucial in analog circuit design to pinpoint the most suitable operating region. To address this gap, the present study focuses on applying this approach to graphene-based devices, scrutinizing the performance of both single-gate (SG) GNR-FET and double-gate (DG) GNR-FET. This analysis includes a comprehensive assessment of the trade-off between transistor efficiency and unit frequency gain, detailed in this article.

This study aims to bridge this research gap by examining the various analog and RF performance parameters of GNR-FETs. Subsequently, a comparison is drawn between the optimized single-gate and double-gate GNR-FET devices in terms of various analog/RF performance parameters. To conduct the trade-off analysis of the doped contact GNR-FETs, an in-depth quantum transport method is employed, utilizing the nonequilibrium Green's function (NEGF) formalism. This approach effectively addresses the Schrödinger equation in conjunction with a two-dimensional Poisson's equation within real space, ensuring self-consistency. The NEGF formalism is widely accepted and extensively used for simulating nano-electronic devices, recognized for its notable conformity with experimental outcomes compared to semiclassical methods^32^. The investigation encompasses a range of analog/RF parameters, including transconductance, transconductance generation factor (TGF), output resistance, intrinsic gain, early voltage, gate capacitance, cut-off frequency, transfer frequency product (TFP), gain frequency product (GFP), and gain transfer frequency product (GTFP) for both single-gate and double-gate GNR-FETs. Results show DG GNR-FETs offer improved electrostatic control, mitigating SCEs, exhibiting superior transconductance, efficiency, frequency response, and wider frequency range operation. To explore the utilization of the suggested GNR-FET devices, we have designed a hybrid 6T static random-access memory (SRAM) cell incorporating this technology. The performance of this hybrid SRAM is optimized by varying the capacitance at the source-channel junction of GNR-FETs, while ensuring other device performance metrics remain unaffected. Subsequently, a comparative analysis has been conducted, evaluating the performance of the hybrid 6T-SRAM against conventional SG GNR-FETs and DG GNR-FETs-based 6T-SRAM designs.

## Device structure and simulation methodology

**Figure 1:**
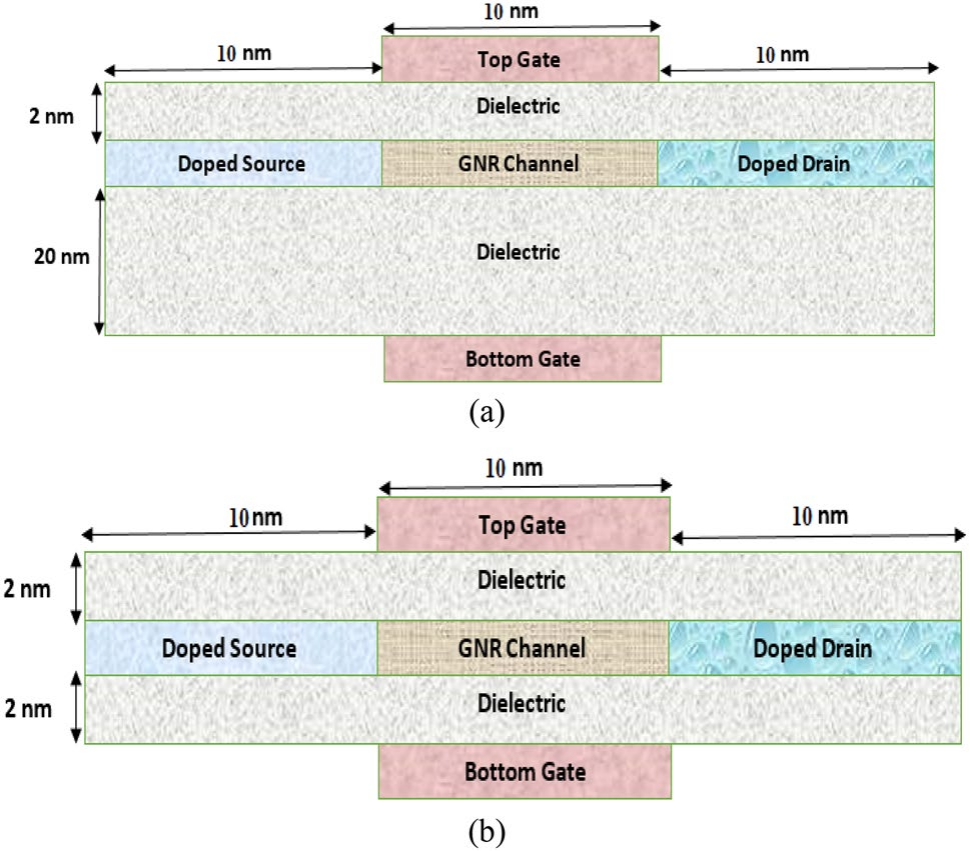
The simulated device structure. (**a**) SG GNR-FET, (**b**) DG GNR-FET.

The simulated n-i-n type single-gate and double-gate device structures, depicted in Fig. 1a,b respectively, consist of a 12-AGNR serving as the channel material, with a band gap of 0.6 eV. The width of GNR channel is 1.37 nm, while source, drain, and gate regions of the device have a length of 10 nm.

The Al_2_O_3_ is considered a top and bottom gate dielectric having a dielectric constant of 10. The source and drain regions are doped with the same density of 2.5 × $10^{13}$ ${\text{cm}}^{ - 2}$ in order to achieve an n-type extension of GNR channel. In case of the double-gate GNR-FET, gate oxide has a physical thickness of 2 nm on both the top and bottom layers. However, for single-gate GNR-FET, bottom gate oxide is ten times thicker than top gate oxide thickness (2 nm) to eliminate gate effect in the device. This approach has been previously employed in^33^. The drain is biased at a voltage of 0.5 V, and all simulations are conducted using the atomistic device simulator NanoTCAD ViDES^34^ at a temperature of 300 K.

The GNR-FET simulation was conducted using the NEGF framework. The crucial aspect of the NEGF formalism involves determining the appropriate Hamiltonian for the material. The tight-binding approximation has been used for the result analysis because it provides reasonable accuracy for the material that has been used during our analysis. In addition, incorporating a *p*_z_ orbital basis set further refines the model by considering a specific orbital shape and symmetries that is relevant to the electronic structure of the material. For simulation purposes, the utilized Hamiltonian is a 2-band model, and it can be expressed as follows^35^:

1 $$ H\left( k \right) = \left[ {\begin{array}{*{20}c} {E_{B} } & {tf\left( k \right)} \\ {tf\left( k \right)^{*} } & {E_{A} } \\ \end{array} } \right] $$

the parameters E_A_ and E_B_ indicate the energy levels situated at the uppermost point of the valence band and the lowermost point of the conduction band, respectively. These parameters are related by the equation *E*_*B*_ − *E*_*A*_ = *E*_*G*_, where *E*_*G*_ denotes the bandgap. The tight-binding Hamiltonian matrix is utilized in the 1-D real space basis of the elementary cell, possesses a value of lateral hopping energy, *t* =  − 2.7 eV^36,37^.

the *f*(*k*) is expressed as^38^:

2 $$ f\left( k \right) = \left( {1 + e^{{ - jk_{y} a_{1} }} + e^{{ - jk_{y} (a_{1} + a_{2} )}} } \right) $$

where a_1_ and a_2_ are the primitive lattice vectors.

Once the Hamiltonian matrix is defined, the next step involves calculating the Green's function, as shown in^39^:

3 $$ G\left( E \right) = \left[ {EI - H - \sum_{S} - \sum_{D} } \right]^{ - 1} . $$

To incorporate the influence of the source and drain contacts, corresponding self-energy matrices $\sum_{S}$ and $\sum_{D}$ are added to the Hamiltonian matrix. The self-energies for the Green’s function are determined through a recursive relation^40^. The computation of the retarded Green’s function involves applying Gaussian elimination to both the Hamiltonian matrix and the self-energy matrices. This allows us to find the charge density on atomic sites for different *k*_*y*_ values using the retarded Green's function, following a similar approach as described in reference^41^. To discretize and solve the partial differential equations (PDEs), numerical methods such as finite difference method has been approximated by the simulator. The quantum mechanical effects such as tunneling and ballistic transport are being taken care by the Non-Equilibrium Green's Function. To determine the charge density, a self-consistent equation is solved alongside the 2-D Poisson equation. The calculations are repeated iteratively until a specific convergence criterion is met. The finite difference method is used to handle the 2-D Poisson equation in the xz plane. Within this iterative inner nonlinear loop, which connects potential energy to charge density, the Newton–Raphson method is utilized^42^. Once convergence is achieved, the source- to-drain current is calculated by evaluating the transmission function through the Landauer formula^39^.

## Results and discussion

**Figure 2:**
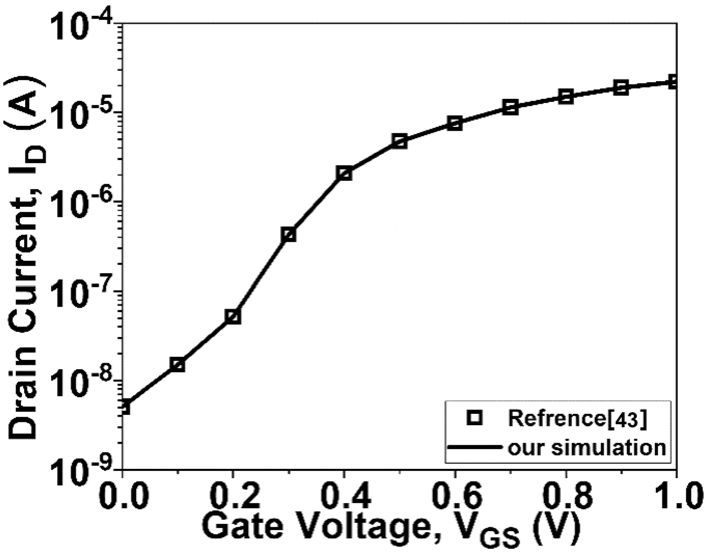
I_D_–V_GS_ characteristics of the simulator and reported^43^ data.

Before initiating the analysis of GNR-FETs with different structure, the accuracy of the current simulator is assessed. To achieve this, a simulation is conducted based on a previous study^43^, focusing on n-i-n GNR-FET devices. The results are depicted in Fig. 2, highlighting the excellent agreement between our simulations and the findings reported in the referenced article.

**Figure 3:**
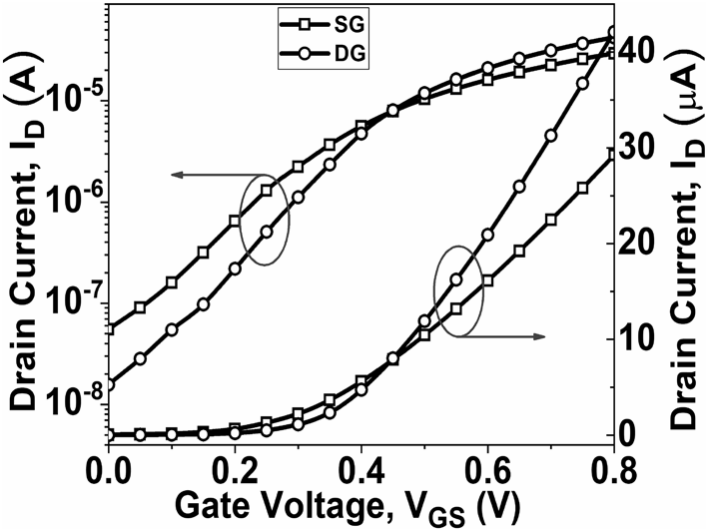
I_D_–V_GS_ of SG GNR-FET and DG GNR-FET devices at *V*_*DS*_ = 0.5 V.

**Figure 4:**
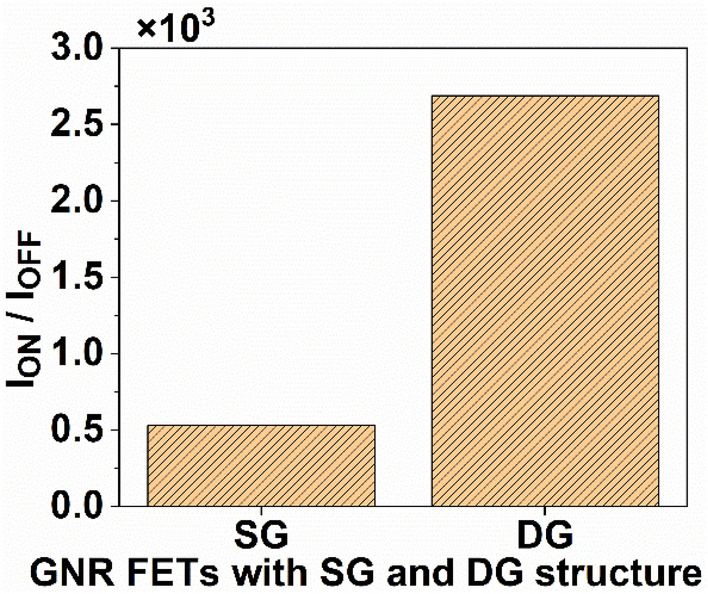
Variation of current ON/OFF ratio with GNR-FETs having SG and DG structure.

After confirming the accuracy of our simulation, we employed the previously explained approach to determine the drain current by varying *V*_*GS*_, as shown in Fig. 3. It is observed from Fig. 3 that the DG GNR-FET exhibits a greater ON current in contrast to that of the SG GNR-FET. The increased ON current in the DG GNR-FET compared to the SG GNR-FET can be attributed to the superior control of channel conductance by the gate in the DG structure. This enhanced control is a result of the gate’s ability to modulate the channel from both the top and bottom, effectively optimizing the flow of charge carriers through the channel. Consequently, the DG configuration provides a more efficient pathway for current to flow, leading to higher ON current levels^44^. When the gate voltage varies from 0 to 0.8 V of *V*_*DS*_, the current changes quickly; hence, the current ON/OFF ratio (*I*_*ON*_/*I*_*OFF*_) is observed as 5.28 × 10^2^ and 2.69 × 10^3^ for SG GNR-FET and DG GNR-FET, respectively, as illustrated in Fig. 4. Here, the ON current is set at *V*_*DS*_ = 0.5 V and *V*_*GS*_ = 0.8 V.

**Figure 5:**
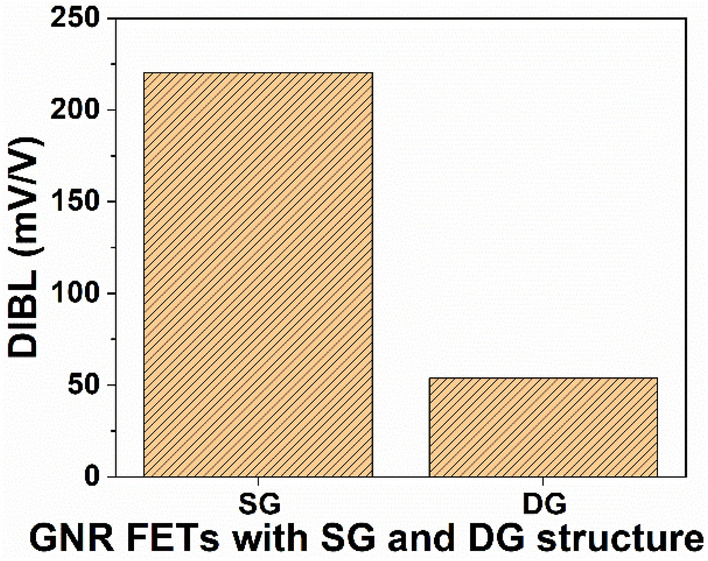
Variation of DIBL with GNR-FETs having SG and DG structure.

Drain induced barrier lowering (DIBL) is a crucial aspect of short-channel effects and serves as a significant consideration in the design of low-power integrated circuits (ICs). As depicted in Fig. 5, it is noted that the DIBL is 220.25 mV/V for SG GNR-FETs. However, this value decreases to 53.74 mV/V for DG GNR-FETs. The higher DIBL in SG structure is due to the higher *I*_*OFF*_, however for the DG structure, the DIBL diminishes, leading to a decrease in *I*_*OFF*_.

**Figure 6:**
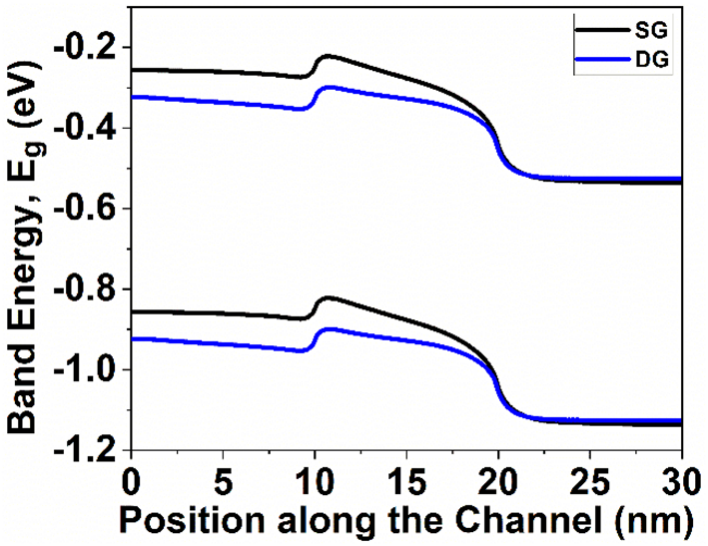
Energy band gap of the GNR-FETs at ON state.

**Figure 7:**
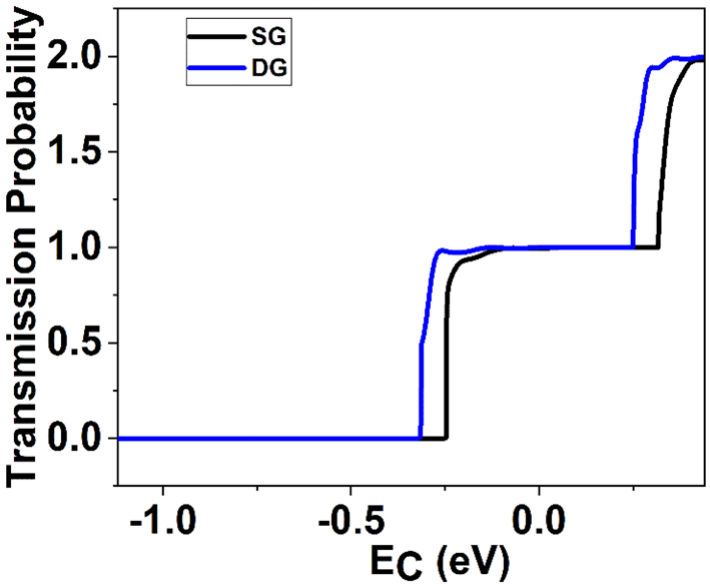
Variation of transmission probability with energy, E (eV) at ON state.

Figure 6 illustrates conduction band profile of the device at ON state. It is observed from Fig. 6 that GNR-FET with DG structure covers lower energy path; therefore, the device with DG structure has higher *I*_*ON*_ compared to GNR-FET with SG structure. This difference in the energy band diagram can be attributed to the higher capacitance in the double gate structure, stemming from the reduced oxide thickness and the presence of two gates in close proximity, as depicted in Fig. 12. The heightened capacitance leads to increased charge inversion at saturation in Field-Effect Transistors, where the channel is fully formed, and the inversion charge reaches its peak^45^. Typically, the distribution of inversion capacitance is higher at the source side due to channel length modulation effects. Consequently, the elevated inversion charge induced by channel length modulation results in a more pronounced shift in the Fermi level at the source side of the double gate structure^46^. Figure 7 shows and transmission probability for the ON-state along device's transport length. It is observed from Fig. 7 that transmission in conduction band increases as wire thickness decreases. The ballistic current is calculated by comparing the transmission with energy. Notably, the transmission steps exhibit dependence on channel and exhibit regions of high transmission at an energy of E = 0.44 eV. The energy observed in SG and DG GNR-FETs is approximately 1.979 eV and 1.996 eV, respectively. Consequently, DG GNR-FET exhibits 1.0086 times higher transmission compared to SG GNR-FET devices.

### Analog performance

Following the analysis of the transfer characteristics of the devices, this section examines the additional significant figures-of-merit (FoMs) for analog integrated circuits. These FoMs include transconductance (*g*_*m*_), transconductance generation factor (TGF), output conductance (*g*_*d*_), output resistance (*r*_0_), early voltage (*V*_*EA*_) and intrinsic gain (*A*_*v*_).

The ability of a device to amplify a signal is determined by its transconductance. A higher transconductance value indicates a greater capacity for amplification or gain. The following definitions and calculations apply to the *g*_*m*_, TGF and *g*_*d*_ parameters^47,48^:

4 $$ g_{m} = \frac{{\partial I_{D} }}{{\partial V_{GS} }} $$

5 $$ TGF = \frac{{g_{m} }}{{I_{D} }} $$

6 $$ g_{d} = \frac{{\partial I_{D} }}{{\partial V_{DS} }}. $$

**Figure 8:**
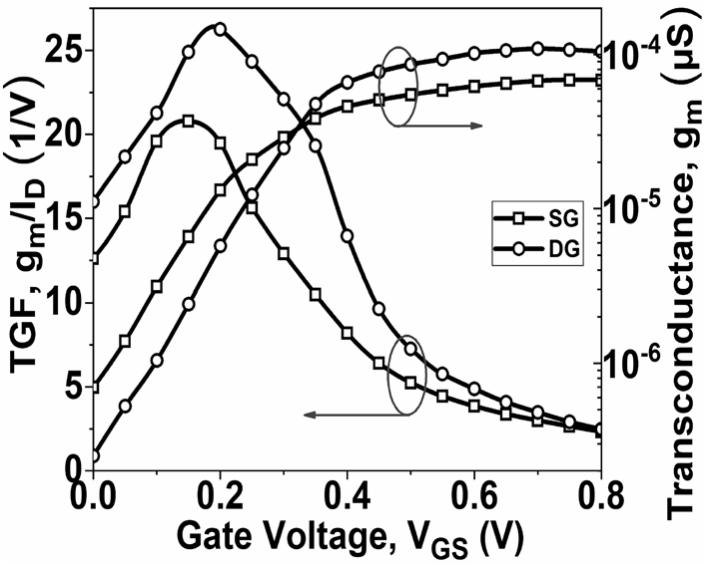
Plot of g_m_ and TGF with *V*_*GS*_ at V_DS_ = 0.5 V.

**Figure 9:**
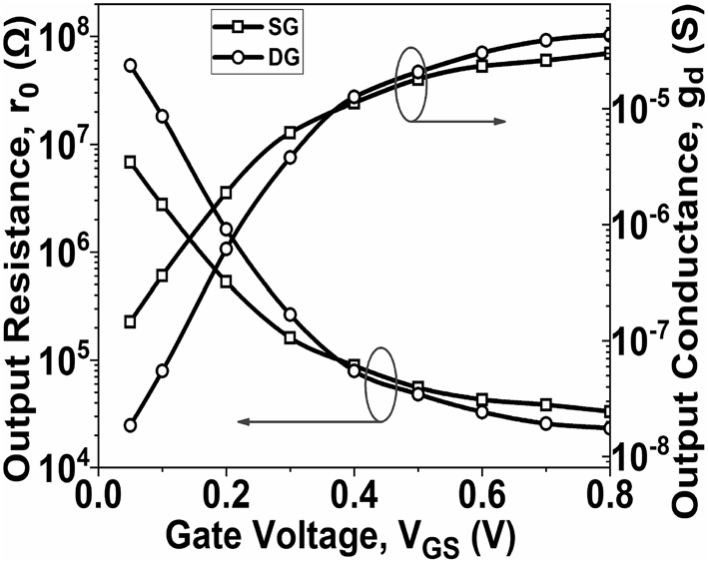
Plot of *r*_0_ and *g*_*d*_ with *V*_*GS*_ at V_DS_ = 0.5 V.

Figure 8 demonstrates the initial increase of *g*_*m*_ with *V*_*GS*_, eventually reaching its peak value. The DG GNR-FET achieves a peak *g*_*m*_ value of 108.92 µS, whereas the SG GNR-FET reaches a maximum value of 68.47 µS. This disparity can be attributed to the proportional relationship between *g*_*m*_ and *I*_*D*_. The TGF signifies the efficient utilization of drain current to attain a desirable *g*_*m*_ value. A higher TGF value implies that the device is suitable for amplifier designs, especially in situations requiring low power. Figure 5 illustrates the variation of TGF for both SG GNR-FET and DG GNR-FET. It is observed that the maximum TGF value is attained at low V_GS_, but degrades significantly as V_GS_ increases, indicating a high gain with minimal power dissipation. Figure 9 illustrates the plot of *r*_0_ and *g*_*d*_ as *V*_*GS*_ varies. The results demonstrate that both the considered devices exhibit a decrease in *r*_0_ and an increase in *g*_*d*_. However, the SG GNR-FET achieves a higher maximum output resistance compared to the DG GNR-FET. Conversely, the DG GNR-FET attains a higher maximum *g*_*d*_ value compared to the SG GNR-FET. A larger *g*_*d*_ value indicates a superior conversion efficiency from drain current to drain voltage.

The intrinsic gain and early voltage can be determined as^46,48^:

7 $$ V_{EA} = {{I_{D} } \mathord{\left/ {\vphantom {{I_{D} } {g_{d} }}} \right. \kern-0pt} {g_{d} }} = I_{D} r_{0} $$

8 $$ A_{v} = g_{m} r_{0} . $$

**Figure 10:**
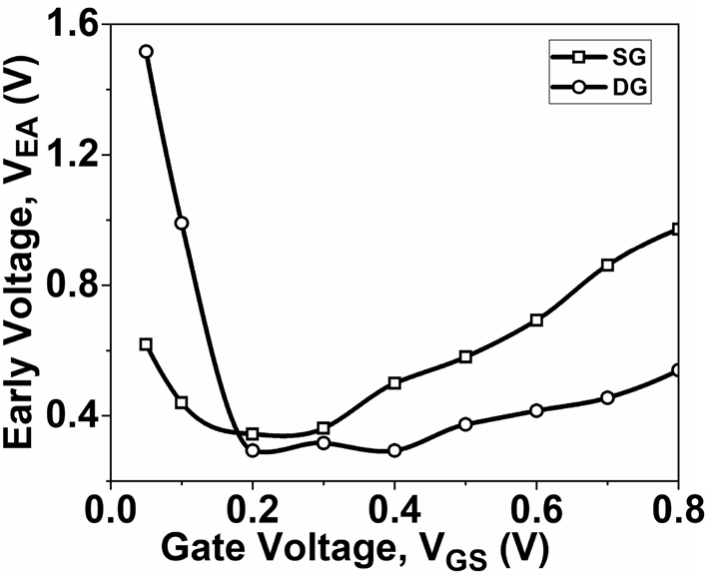
Plot of V_EA_ as a function of V_GS_ at V_DS_ = 0.5 V.

**Figure 11:**
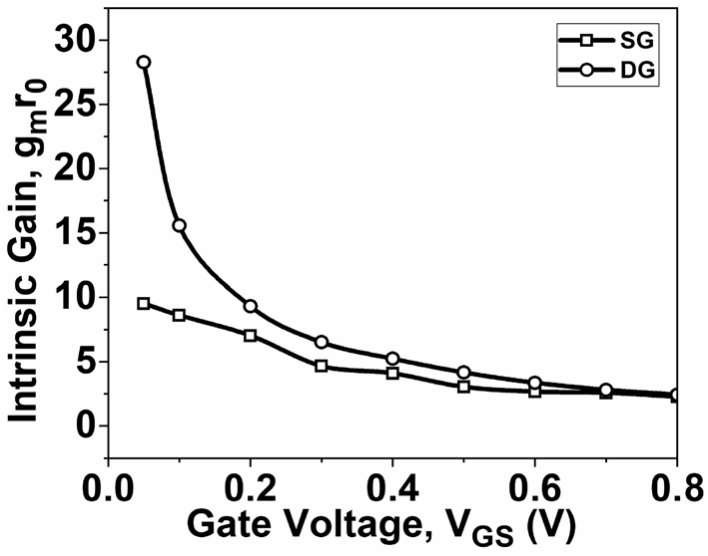
Plot of *A*_*v*_ as a function of V_GS_ at V_DS_ = 0.5 V.

Figures 10 and 11 illustrate the relationship between *V*_*EA*_ and *V*_*GS*_, as well as the *A*_*v*_ relation with respect to *V*_*GS*_, respectively. These relationships are determined by Eqs. (7) and (8) respectively. To achieve better analog performance, it is desirable to have higher values of *V*_*EA*_ and *A*_*v*_. It can be observed from Fig. 10 that the maximum value of *V*_*EA*_ is obtained with DG GNR-FET. Consequently, the *A*_*v*_, which is the product of *g*_*m*_ and *r*_0_, is primarily influenced by *r*_0_ at both low *V*_*GS*_ and high *V*_*GS*_. As a result, the maximum value of *A*_*v*_ is attained with DG GNR-FET, as illustrated in Fig. 11.

### RF performance

This section focuses on conducting an analysis of the RF performance for both SG and DG GNR-FET devices. When it comes to RF analysis, the two primary FoMs considered are the gate capacitance (*C*_*G*_) and cut-off frequency (*f*_*T*_).

**Figure 12:**
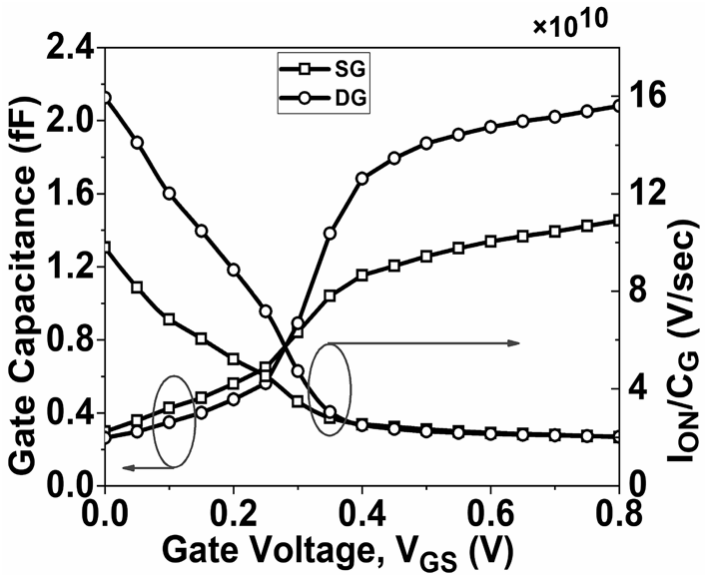
Plot of C_G_ and I_ON_/C_G_ as a function of V_GS_.

The gate capacitance of the device is crucial when dealing with RF applications. It is determined by calculating the ratio of the change in the concentration of charge carriers to the change in voltage^46^. Figure 12 depicts the gate capacitance (*C*_*G*_) for both SG and DG GNR-FET devices. The DG GNR-FET has a peak *C*_*G*_ value of 2.08 fF, while the SG GNR-FET has a peak value of 1.45 fF. The *I*_*ON*_*/C*_*G*_ plot is depicted in Fig. 12. A greater *I*_*ON*_*/C*_*G*_ ratio indicates a faster switching operation in the device. Additionally, it is noted that the highest *I*_*ON*_*/C*_*G*_ ratio occurs at low *V*_*GS*_, but as *V*_*GS*_ increases, the *I*_*ON*_*/C*_*G*_ ratio decreases significantly.

**Figure 13:**
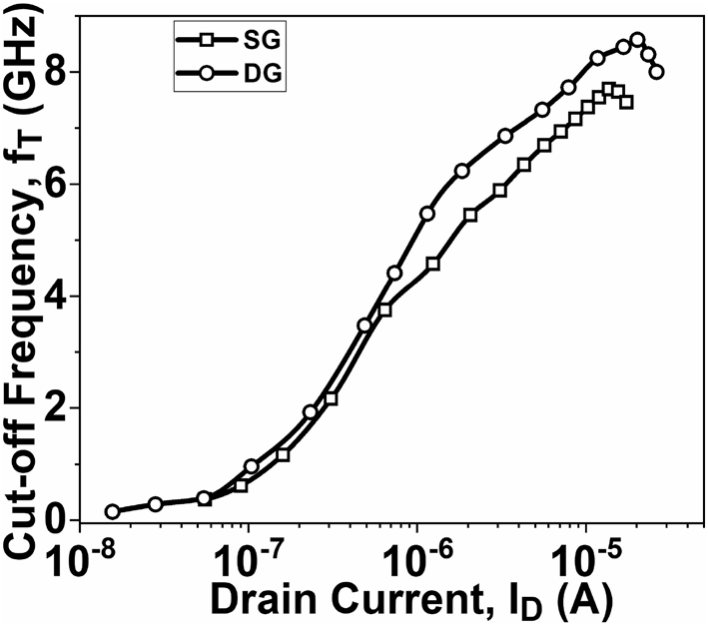
Plot of f_T_ as a function of I_D_.

In RF applications, the cut-off frequency (*f*_*T*_ = *g*_*m*_ / 2π *C*_*G*_) is an important FoM for a device. It is the frequency at which the current gain reaches unity and is shown in Fig. 13. According to the equation, a device with a higher gm by *C*_*G*_ value will result in a larger cut-off frequency. As a result, the DG GNR-FET exhibits a sharper cut-off frequency compared to the SG GNR-FET.

**Figure 14:**
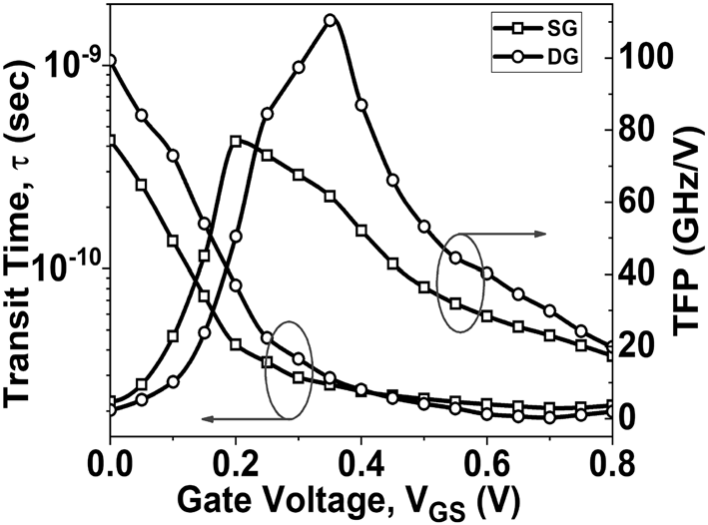
Variation of τ and TFP as a function of V_GS_.

The transit time (*τ* = 1/2*π f*_*T*_) is an important factor to consider in RF analysis^49^. The transit time represents the duration required for charge carriers to move from the source to the channel. The variation of *τ* with respect to *V*_*GS*_ is illustrated in Fig. 14. It can be observed from the figure that the *τ* value decreases for both SG and DG GNR-FETs. This decrease in *τ* is attributed to the higher *f*_*T*_ of the device, which is desirable for enhanced switching performance.

The design of analog circuits requires careful consideration of the relationship between device efficiency, intrinsic gain, and bandwidth. Determining the optimal operating point involves a trade-off analysis that takes into account parameters such as transconductance frequency product (TFP), gain frequency product (GFP), and gain transconductance frequency product (GTFP)^48,50^. The complexity arises from the diverse factors influencing these parameters, including transistor construction, circuit design, and external elements. The relationships between these factors are nonlinear and interdependent, posing challenges in optimization. Balancing conflicting objectives, such as maximizing gain while maintaining efficiency, requires a deep understanding of semiconductor physics and optimization techniques, often involving iterative processes.

**Figure 15:**
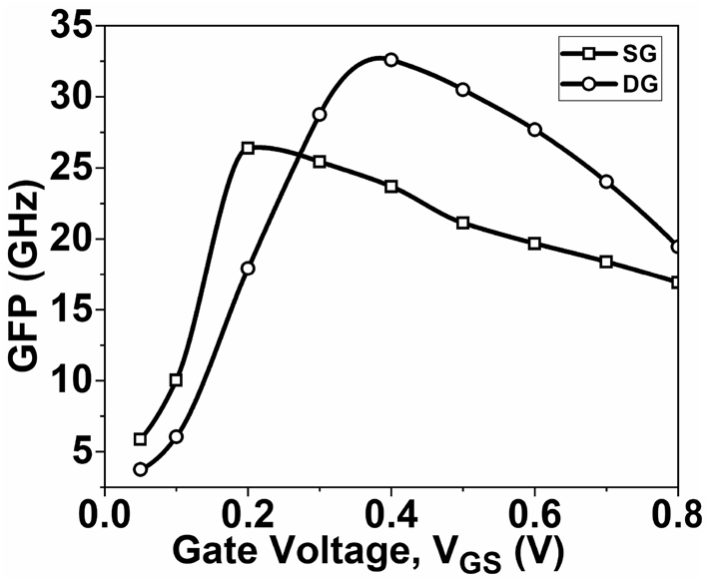
Variation of GFP as a function of V_GS_.

TFP is calculated by multiplying the TGF and frequency (*f*_*T*_) and captures the trade-off between power and bandwidth in moderate to high-speed circuit designs^51^. A higher TFP value allows the circuit designer to fine-tune the device's performance by adjusting the trade-off between transconductance and cut-off frequency. Figure 14 shows the plot of TFP as a function of *V*_*GS*_. It is observed that the DG GNR-FET exhibits a higher TFP value compared to the SG GNR-FET. The GFP represents the trade-off between gain and frequency, particularly in high-frequency applications of operational amplifiers^48^. The GFP is the product of intrinsic gain and *f*_*T*_*.* Figure 15 displays the plot of GFP as a function of *V*_*GS*_. Both the SG GNR-FET and DG GNR-FET show a high TFP value at low *V*_*GS*_, but this value decreases as *V*_*GS*_ increases. However, the DG GNR-FET device reaches a higher peak value compared to the SG GNR-FET device.

**Figure 16:**
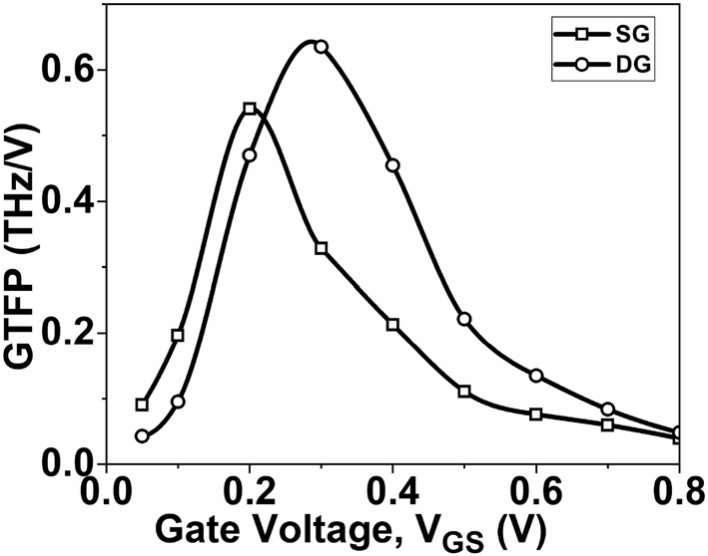
Variation of GTFP, as a function of V_GS_.

Figure 16 shows the plot of GTFP as a function of *V*_*GS*_. It is observed from Fig. 16 that the DG GNR-FET exhibits a higher GTFP value compared to the SG GNR-FET. The GTFP is a figure of merit that indicates the performance of an amplifier in terms of gain, transistor efficiency and frequency response. This provides the circuit designer with the flexibility to select the optimal operating region by balancing factors such as gain, transconductance, and speed.

### Analysis of 6T-SRAM cell design

**Figure 17:**
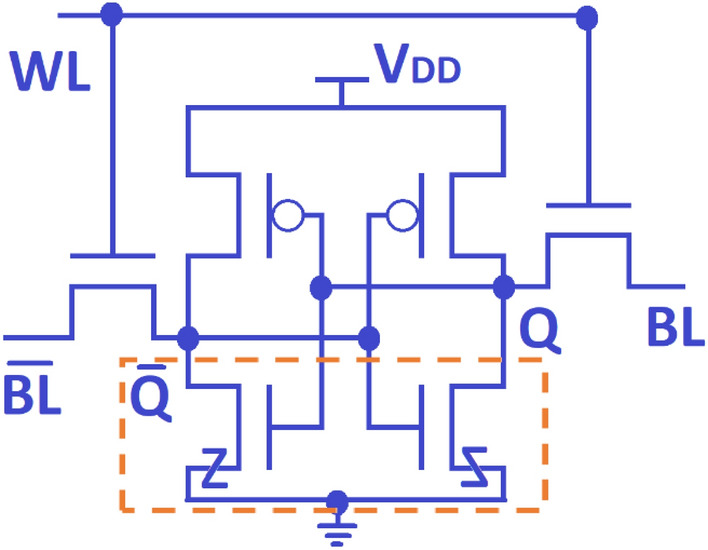
6T-SRAM designed with two cross-coupled inverters and two access transistors.

The Fig. 17 demonstrates the widely used and basic design of a symmetric six-transistor (6T) structure, which serves as the fundamental topology for CMOS SRAM cells. At the heart of this structure are two interconnected inverters which retain a distinct logical state in the Q and ${\overline{\text{Q}}}$ nodes. These nodes can be interacted with either ‘read’ or ‘written to’ by using the bit-lines (BLs) via the access transistors (ATs), contingent upon the signal from the word-line (WL). Otherwise, the voltage levels at nodes Q and ${\overline{\text{Q}}}$ remain constant during the hold state.

**Figure 18:**
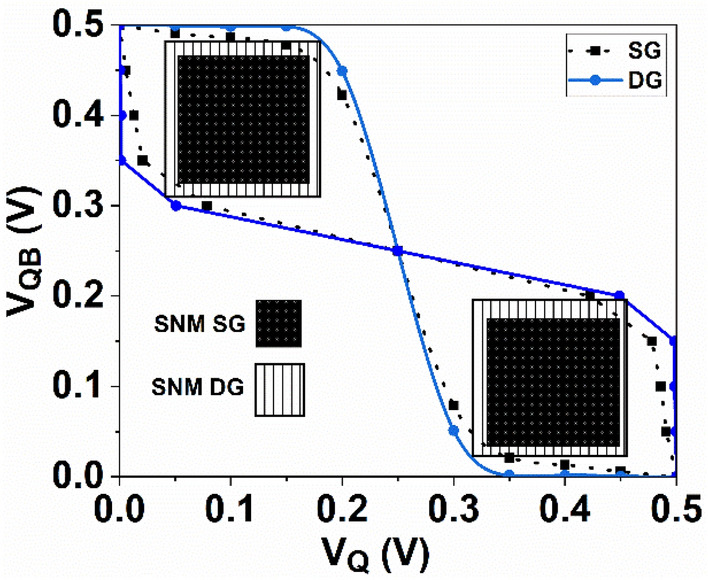
Butterfly curve for the 6T-SRAM cell with SG and DG GNR-FETs.

The stability of SRAM is commonly assessed through its static noise margin (SNM), which indicates the maximum level of noise voltage at the inverters output that the SRAM can withstand without altering the memory cell’s contents. SNM is graphically determined from the butterfly curves of the SRAMs. It is defined as the minimum length of the sides of the two largest squares that can fit within the two lobes of the butterfly curve, as illustrated in Fig. 18. Figure 18 demonstrates that the hold SNM is superior in DG SRAM compared to conventional SRAM based on SG. This improvement in hold SNM is attributed to the rapid output transition in the hybrid SRAMs.

## Conclusion

This paper focuses on evaluating the impact of single-gate (SG) and double-gate (DG) structures on the analog/RF figures-of-merit (FoMs) of graphene nanoribbon field-effect transistors (GNR-FETs). The research demonstrates that the double-gate structure exhibits significantly superior analog/RF figures-of-merit (FoMs) compared to the single-gate structure. The DG GNR-FET demonstrates a current ON/OFF ratio that is 409% higher than the SG GNR-FET. Additionally, the transconductance, transconductance generation factor, and intrinsic gain of the DG GNR-FET are 59.1%, 26.2%, and 197% superior to those of the SG GNR-FET, respectively. The study also reveals significant changes in RF FoMs. The cut-off frequency of the DG GNR-FET device is 11.4% higher than that of the SG GNR-FET device. Furthermore, the TFP, GFP and GTFP are 43.7%, 23.5% and 17.5% higher, respectively, compared to the SG device structure. These improvements can be attributed to the superior electrostatic control of the channel provided by the double-gate structure. These results hold significant value for the design of nanoscale devices intended for high-frequency applications. Based on these findings, it is suggested that the DG GNR-FET structure is more prominent when considering the analog/RF performance evaluation of the device.

## Data Availability

The data that support the findings of this study are available from the corresponding author, [akram14407@gmail.com], upon reasonable request.
